# The Impact of Statins on the Survival of Patients with Advanced Hepatocellular Carcinoma Treated with Sorafenib or Lenvatinib

**DOI:** 10.3390/cancers16020249

**Published:** 2024-01-05

**Authors:** Ji Eun Han, Jisu Kim, Jae Youn Cheong, Soon Sun Kim, Sun Gyo Lim, Min Jae Yang, Choong-Kyun Noh, Gil Ho Lee, Jung Woo Eun, Bumhee Park, Hyo Jung Cho

**Affiliations:** 1 Department of Gastroenterology, Ajou University School of Medicine, Suwon 16499, Republic of Korea; 110518@aumc.ac.kr (J.E.H.); jaeyoun620@aumc.ac.kr (J.Y.C.); cocorico99@aumc.ac.kr (S.S.K.); mdlsk75@aumc.ac.kr (S.G.L.); creator1999@aumc.ac.kr (M.J.Y.); cknoh23@aumc.ac.kr (C.-K.N.); micorie@aumc.ac.kr (G.H.L.); jetaimebin@aumc.ac.kr (J.W.E.); 2 Office of Biostatistics, Medical Research Collaborating Center, Ajou Research Institute for Innovative Medicine, Ajou University Medical Center, Suwon 16499, Republic of Korea; k.jisu5107@gmail.com (J.K.); bhpark@aumc.ac.kr (B.P.); 3 Department of Biomedical Informatics, Ajou University School of Medicine, Suwon 16499, Republic of Korea

**Keywords:** statin, multityrosine kinase inhibitor, sorafenib resistance, hepatocellular carcinoma, lipophilic statin, hydrophilic statin

## Abstract

**Simple Summary:**

Hepatocellular carcinoma (HCC), the most prevalent form of primary liver cancer, is a significant cause of cancer mortality. Patients with advanced HCC commonly receive systemic therapy, including tyrosine kinase inhibitors (TKIs) such as sorafenib and lenvatinib. However, TKI resistance remains a challenge. Statins, known for their lipid-lowering properties, also show potential anti-cancer effects, particularly in HCC, by inhibiting the mevalonate pathway. Evidence suggests statins may reduce HCC risk in patients with certain conditions and potentially enhance the efficacy of TKIs. Our study, utilizing data from Korea’s Health Insurance Review and Assessment Service (HIRA), investigated the clinical benefits of statins in patients with advanced HCC treated with TKIs, verifying the timing, type, and dosage of statins and their impact on patient survival outcomes.

**Abstract:**

We aimed to evaluate the survival benefits of coadministering statins and multityrosine kinase inhibitors (TKIs) in patients with advanced hepatocellular carcinoma (HCC). Data from the Health Insurance Review and Assessment Service in Korea (2010–2020) were utilized. Statin use (≥28 cumulative defined daily doses) was analyzed, with 1534 statin users matched to 6136 non-users (1:4 ratio) using propensity scores. Primary and secondary outcomes were overall survival (OS) and progression-free survival (PFS). Statin use significantly improved OS (hazard ratio [HR] 0.77, 95% confidence interval [CI] 0.72–0.82, *p* < 0.001) and PFS (HR 0.78, 95% CI 0.74–0.84, *p* < 0.001). Continuous or post-TKI statin users had better OS, while discontinuation after TKI use led to poorer OS. Both lipophilic and hydrophilic statins improved OS and PFS, particularly with ≥730 cumulative defined daily doses. In conclusion, combining statins and TKIs in patients with advanced HCC yielded significant survival benefits, influenced by statin dosage and duration. Continuous statin administration post-TKI treatment is crucial for improving outcomes in patients with HCC.

## 1. Introduction

Hepatocellular carcinoma (HCC), the most common type of primary liver cancer, is one of the leading causes of cancer mortality worldwide [1]. Most patients with HCC are at an advanced stage at the time of diagnosis, and systemic therapy is the only feasible treatment modality at this stage [2].

Sorafenib and lenvatinib, multityrosine kinase inhibitors (TKIs), were used as first-line treatment for patients with advanced HCC for almost 10 years, until immune checkpoint inhibitor-based immunotherapy was introduced [3,4]. TKIs are still considered as an alternative for patients with contraindications to atezolizumab plus bevacizumab (Atezo+Bev) therapy or for sequential therapy after Atezo+Bev treatment failure. In a previous study, although TKIs resulted in improved overall survival (OS) compared to that with placebo, the survival benefit on average was 3 months [3,5]. Thus, resistance to TKIs has been a major challenge in the systemic treatment of HCC [6].

Statins are competitive inhibitors of 3-hydroxy-3-methylglutaryl-CoA reductase (HMG-CoA), and they also have potential chemopreventive and cytotoxic effects on cancer cells in different types of cancers, primarily through the inhibition of the mevalonate pathway, independent of their lipid-lowering effect [7,8,9,10]. Statin use may be associated with a lower risk of HCC development in patients with hepatitis B or C infection and diabetes mellitus (DM) [11,12,13,14,15,16]. Moreover, studies that included patients undergoing liver resection for very early and early HCC (BCLC-0 and A) have demonstrated improved recurrence-free survival for patients under statin therapy [17,18]. Notably, statins not only mitigate the risk of HCC development but also exert direct effects on cancer cells. Statins exhibit direct effects on HCC cells by the inhibition of cell growth and the induction of apoptosis. Emerging molecular evidence suggests that statins might potentiate the anticancer effects of TKIs, specifically in chronic myeloid leukemia, non-small cell lung cancer (NSCLC), renal cell cancer, and head and neck squamous cell cancer (HNSCC) [19,20,21,22]. Additionally, retrospective cohort studies verified that statins could improve clinical outcomes in patients with NSCLC and HNSCC treated with TKIs [23,24].

The Health Insurance Review and Assessment Service (HIRA) in Korea is responsible for the claims review and quality assessment of the National Health Insurance (NHI). The HIRA research database provides information on age, sex, codes for diagnosis, prescribed drugs, and treatment, including surgical history and procedures.

We aimed to verify the potential clinical benefits of statins in patients with advanced HCC treated with sorafenib or lenvatinib by analyzing large-scale data from HIRA in Korea. We also investigated the impact of the timing of statin administration (pre-TKI use, continuous use, and post-TKI use) and optimal statin type and dose on the survival outcomes of these patients.

## 2. Materials and Methods

### 2.1. Data Source

More than 98% of Koreans are obligated to join the NHI Service. The HIRA is a national institution that reviews and evaluates medical costs and the quality of medical care. In the present study, information from the HIRA database, including data on patient demographics, prescriptions, treatments, and diagnoses, was reviewed. The study was conducted in accordance with the Declaration of Helsinki and approved by the Institutional Review Board of Ajou University Hospital (AJIRB MED-EXP-2021-552). The requirement for prior consent was waived because of the retrospective nature of the study. De-identification processing was performed, and the data were approved by the national health information data request review committee of HIRA.

### 2.2. The Study Population and Definition of Terms

**Figure 1:**
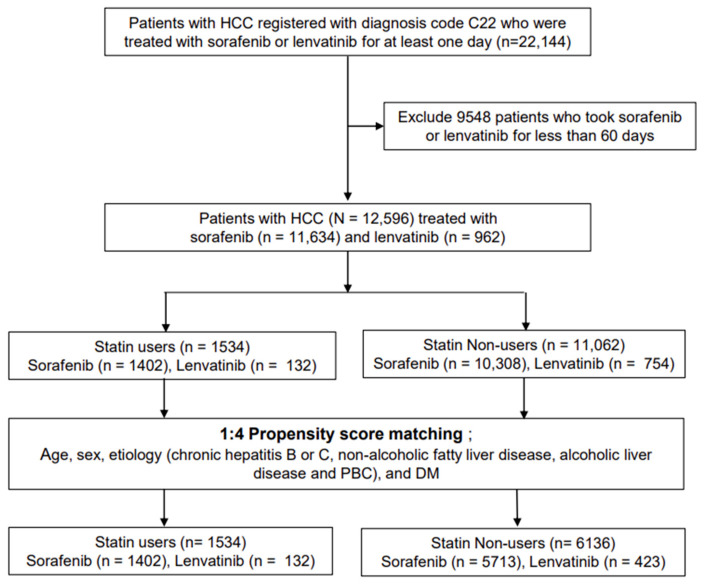
Flow chart of the study population. DM, diabetes mellitus; PBC, primary biliary cholangitis. In terms of statin administration timing, pre-TKI statin use was defined as statin use before TKI treatment with discontinuation after TKI treatment initiation. Post-TKI use was defined as statin administration initiated after TKI administration, given concurrently for more than 30 days. Continuous statin use was defined as statin use from the period before TKI treatment to the period after TKI treatment (Figure 2) Lipophilic statins included lovastatin, simvastatin, atorvastatin, fluvastatin, and pitavastatin, whereas hydrophilic statins included pravastatin and rosuvastatin. Regional distribution was subclassified to determine urban–rural differences in the survival outcomes of patients; urban regions included Seoul, Gyeonggi province, and Special cities, whereas others were classified as rural regions.

Overall, 22,144 patients, aged ≥18 years, who were diagnosed with HCC and took sorafenib or lenvatinib for more than 1 day between 1 January 2010 and 31 December 2020 were initially screened. Considering that the treatment response is evaluated every 2–3 months in patients with advanced HCC treated with sorafenib or lenvatinib, we defined sorafenib- or lenvatinib-treated patients as those who had received TKIs for >60 days without interruption. Patients who received sorafenib or lenvatinib for <60 days (*n* = 9548) were excluded. Thus, 12,596 patients were included in the analysis. Statin dose was measured using the cumulative defined daily dose (cDDD). Statin use was defined as ≥28 cDDDs of filled statin prescriptions and nonuse was defined as <28 cDDDs. Of the 12,596 patients, 11,062 were statin nonusers and 1534 were statin users (Figure 1). Statin dose was subclassified as follows: 28–180 cDDDs, 181–365 cDDDs, 366–730 cDDDs, 731–1095 cDDDs, and ≥1096 cDDDs.

### 2.3. Data Collection

Using the Korea Classification of Disease (KCD), based on the International Classification of Diseases, 11th Revision (ICD-11), we collected codes for all diseases and procedures. Diseases diagnosed within 1 year before HCC diagnosis were identified as comorbidities. We collected anthropometric and demographic data such as age, sex, and region. Clinical data included a history of chronic hepatitis B or C, nonalcoholic fatty liver disease, alcoholic liver disease, or other liver diseases, such as primary biliary cirrhosis (PBC), and comorbidities, such as liver cirrhosis, hypertension (HTN), DM, cerebrovascular disease, and cardiovascular disease. Additional data were collected as follows: history of statin exposure, including the dose and type of statin, timing of statin use, duration of statin administration (cDDD), history of HCC treatment before sorafenib or lenvatinib use, and history of aspirin and antidiabetic medications, such as dipeptidyl peptidase-4 (DPP-4) inhibitor, insulin, metformin, sodium glucose cotransporter-2 (SGLT-2) inhibitor, sulfonylurea, and thiazolidinedione (TZD).

### 2.4. Outcomes

The primary outcome was OS, defined as the time between the index date (first day of TKI treatment) and death from any cause. The secondary outcome was progression-free survival (PFS), defined as the time between the first prescription and last administration of TKIs.

### 2.5. Statistical Analysis

All statistical analyses were performed using R version 4.1.3, R Foundation for Statistical Computing, Vienna, Austria (http://www.r-project.org, accessed on 22 September 2022), with a *p*-value of < 0.05 considered statistically significant. Continuous variables with a normal distribution are expressed as mean ± standard deviation, and categorical variables are expressed as numbers with percentages. Statin users were matched with nonusers in a 1:4 ratio using propensity score matching to balance baseline characteristics and minimize potential confounding. Variables included in propensity score matching were age, sex, etiology (chronic hepatitis B or C, nonalcoholic fatty liver disease, alcoholic liver disease, and PBC), and DM. Kaplan–Meier analysis was performed to compare OS and PFS between groups. Univariate and multivariate Cox regression analyses were performed to identify the risk factors associated with all-cause death and tumor progression. The results are presented as hazards ratios (HRs) with 95% confidence intervals (CIs).

## 3. Results

### 3.1. Comparing Baseline Characteristics between Statin Users and Nonusers in Unmatched and PS-Matched Cohorts

The study compared baseline characteristics between statin users and nonusers in unmatched and propensity score-matched cohorts. Initially, 12,596 participants were involved, and after propensity score matching for age, sex, etiology, and DM, 1534 statin users (including 973 using lipophilic statins and 561 using hydrophilic statins) were matched in a 1:4 ratio with 6136 nonusers. Table 1 summarizes the baseline characteristics of statin users and nonusers. The median follow-up period was 95 months (range: 56–190 months) for statin nonusers and 119.5 months (range: 63–250 months) for statin users. The baseline characteristics revealed that statin users tended to be older and had a higher prevalence of alcoholic liver disease but a lower frequency of chronic hepatitis B virus infection compared to nonusers. Additionally, statin users were more likely to have comorbid conditions such as DM, fatty liver, cirrhosis, PBC, HTN, cardiovascular disease, and cerebrovascular disease. Moreover, a larger proportion of statin users were using aspirin and antidiabetic medications. After propensity score matching, both groups showed no significant differences in the proportion of patients with a history of alcoholic liver disease and DM. The majority of both statin users (91.4%) and nonusers (93.1%) were being treated with sorafenib (Table 1).

### 3.2. Statin Use and Survival Outcome

**Figure 3:**
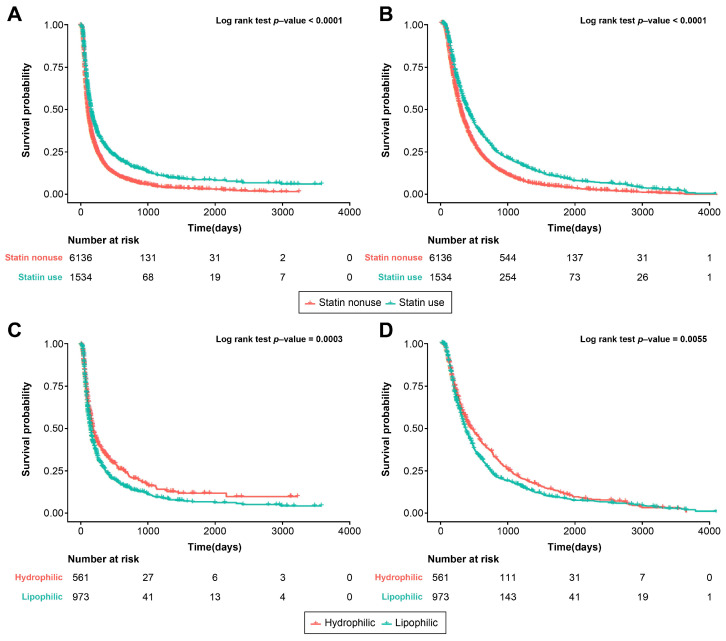
Kaplan–Meier curves of OS and PFS according to statin use and statin type in the PS-matched cohort. (**A**) Comparison of OS between statin users and non-users. (**B**) Comparison of PFS between statin users and non-users. (**C**) Comparison of OS according to statin type (hydrophilic vs. lipophilic). (**D**) Comparison of PFS according to statin type (hydrophilic vs. lipophilic). OS, overall survival; PFS, progression-free survival; PS, propensity score.

Both OS and PFS were significantly better for statin users than for statin nonusers in the PS-matched cohort (log rank *p* < 0.001; Figure 3A,B). The positive impact of statin use was noticeable among patients treated with sorafenib (log rank *p* < 0.001 and *p* = 0.001, respectively; Figure S1A,B), but not in those treated with lenvatinib (shown in Figure S2A,B).

Multivariate Cox regression analysis was performed to identify factors associated with OS and PFS. Statin use was linked to improved OS (HR, 0.77; 95% CI, 0.72–0.82; *p* < 0.001), as were other factors like aspirin, metformin, SGLT-2 inhibitors, sulfonylurea use, and a history of HTN and cardiovascular disease. However, insulin use (HR, 1.24; 95% CI, 1.16–1.33; *p* < 0.001; Table 2) was associated with worse OS. For PFS, factors associated with better outcomes included living in an urban region, having a history of HTN, cardiovascular disease, cerebrovascular disease, and using statins (HR, 0.78; 95% CI, 0.74–0.84; *p* < 0.001), aspirin (HR, 0.63; 95% CI, 0.55–0.73; *p* < 0.001), metformin (HR, 0.72; 95% CI, 0.62–0.84; *p* < 0.001), and sulfonylureas (HR, 0.83; 95% CI, 0.69–1.00; *p* = 0.049). On the other hand, age > 60 years (HR, 1.01; 95% CI, 1.00–1.01; *p* = 0.006) and insulin use (HR, 1.26; 95% CI, 1.18–1.35; *p* < 0.001) were associated with poorer PFS (Table 3).

### 3.3. The Timing of Statin Use and Survival Outcome

In multivariate analysis for OS, it was found that continuous statin use (HR, 0.87; 95% CI, 0.80–0.95; *p* = 0.002) and post-TKI statin use (HR, 0.43; 95% CI, 0.38–0.50; *p* < 0.001) were significantly associated with improved OS. However, pre-TKI statin use (HR, 1.33; 95% CI, 1.14–1.54; *p* < 0.001) was identified as an independent risk factor for poorer OS. Other factors associated with better OS included a history of DM, fatty liver, HTN, cardiovascular disease, and the use of aspirin, metformin, SGLT-2 inhibitors, and sulfonylureas (Table 4). Conversely, insulin use significantly deteriorated OS (HR, 1.23; 95% CI, 1.15–1.32; *p* < 0.001). In the analysis for PFS, only post-TKI statin use (HR, 0.42; 95% CI, 0.38–0.48; *p* < 0.001) significantly improved PFS, while continuous statin use did not (HR, 0.94; 95% CI, 0.87–1.02; *p* = 0.122). Other factors influencing PFS were consistent with those for OS. Factors such as living in an urban region, a history of DM, HTN, cardiovascular disease, and cerebrovascular disease, as well as the use of aspirin, DPP-4 inhibitors, metformin, and sulfonylureas, were associated with better PFS. Pre-TKI statin use (HR, 1.56; 95% CI, 1.35–1.80; *p* < 0.001) and insulin use (HR, 1.25; 95% CI, 1.17–1.33; *p* < 0.001) were identified as independent risk factors for an unfavorable outcome in terms of PFS (Table 5). In summary, the timing of statin use had a significant impact on survival outcomes, with post-TKI statin use showing the most favorable results, while pre-TKI statin use had a detrimental effect on survival. Multiple other factors, including comorbidities and medication use, also influenced survival outcomes.

### 3.4. Statin Type and Survival Outcome

Regarding statin type, hydrophilic statins showed more favorable outcomes in both OS and PFS compared to lipophilic statins (log rank *p* < 0.001 and *p* = 0.006, respectively; Figure 3C,D). Notably, both types of statins led to significant improvements in survival despite significant differences in survival outcomes. For users of lipophilic statins, the HR for PFS was 0.74 (95% CI, 0.69–0.80; *p* < 0.001) and for OS, it was 0.75 (95% CI, 0.69–0.81; *p* < 0.001). For users of hydrophilic statins, the HR for PFS was 0.63 (95% CI, 0.57–0.69; *p* < 0.001), and for OS, it was 0.59 (95% CI, 0.53–0.66; *p* < 0.001; Table S1). This indicates that while both statin types had a positive impact on survival, hydrophilic statins appeared to offer even greater benefits.

### 3.5. Statin Dose and Survival Outcome

**Figure 4:**
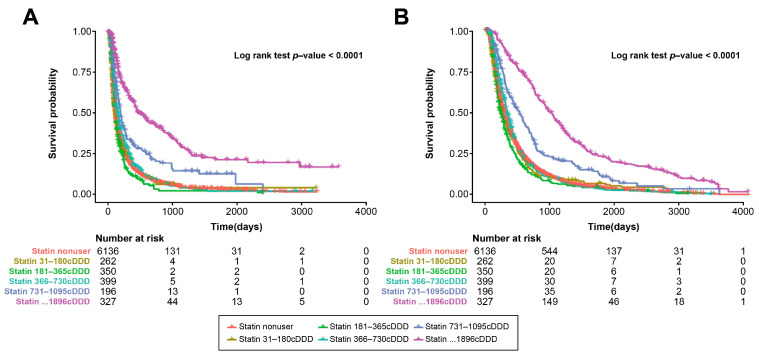
Kaplan–Meier curves for OS and PFS according to cumulative statin dose in PS-matched cohort. (**A**) OS of patients with HCC treated with sorafenib or lenvatinib according to cumulative statin dose. (**B**) PFS of patients with HCC treated with sorafenib or lenvatinib according to cumulative statin dose. HCC, hepatocellular carcinoma; OS, overall survival; PFS, progression-free survival; PS, propensity score.

In the PS-matched cohort, the administration of a high cumulative dose of statins (>730 cDDD) had a substantial positive impact on both OS and PFS. In the group receiving 731 to 1095 cDDD, the HR was 0.46 (95% CI, 0.46–0.69; *p* < 0.001) for tumor progression and 0.48 (95% CI, 0.48–0.75; *p* < 0.001) for all-cause death, both indicating significant benefits (*p* < 0.001). In the group with more than 1096 cDDD, the HR was even lower at 0.34 (95% CI, 0.28–0.41; *p* < 0.001) for tumor progression and 0.34 (95% CI, 0.28–0.42; *p* < 0.001) for all-cause death, underlining a strong association between higher cumulative statin doses and improved survival outcomes (shown in Table S2 and Figure 4). This suggests that higher cumulative doses of statins are linked to more favorable results in terms of survival, emphasizing the importance of the dose in achieving positive effects.

### 3.6. Multivariate Stratified Analysis

**Figure 5:**
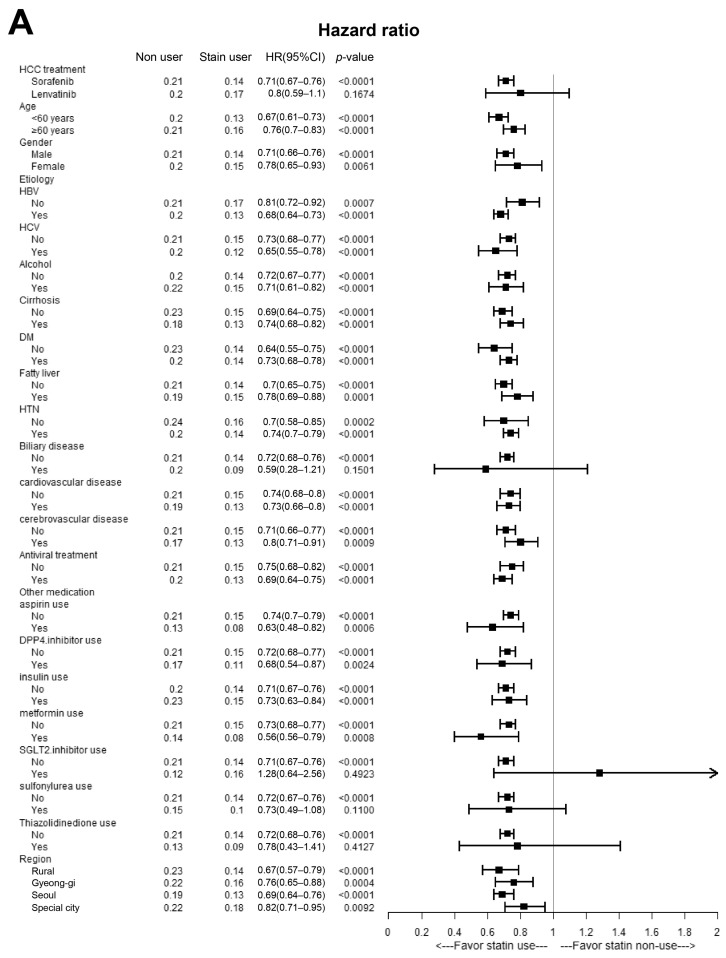

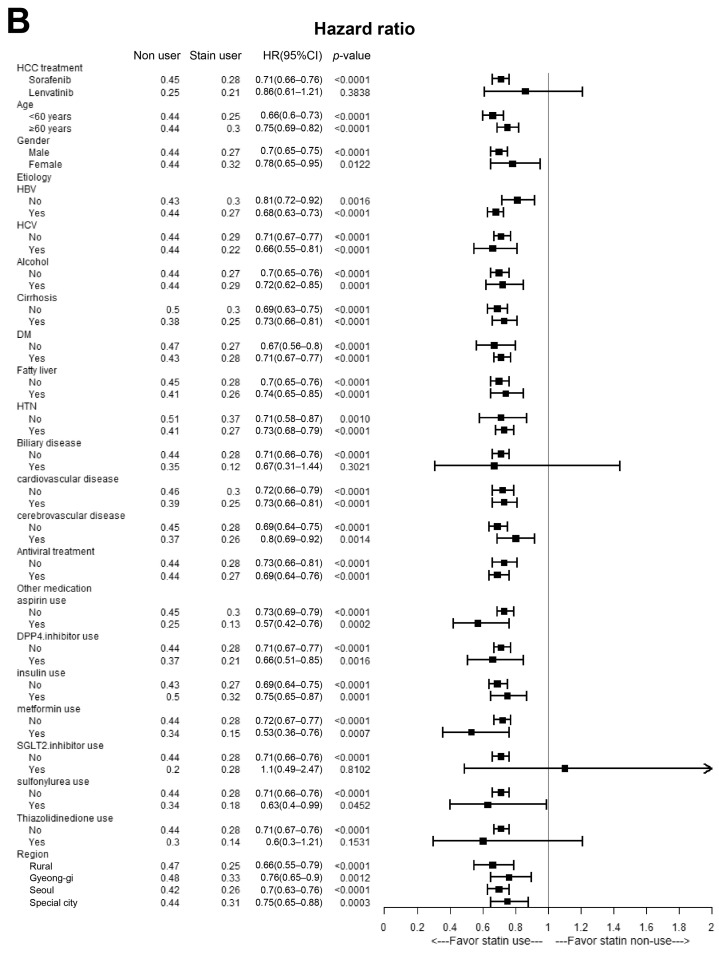
Multivariate stratified analyses for the association between statin usage and OS/PFS in patients with HCC treated with sorafenib or lenvatinib. HR and 95% CI of the difference in mortality and tumor progression risk between statin users and non-users were determined using multivariate Cox regression hazard models based on adjusted covariates. (**A**) Multivariable stratified analyses for the association between statin use and OS in patients with HCC treated with sorafenib or lenvatinib. (**B**) Multivariate stratified analyses for the association between statin use and PFS in patients with HCC treated with sorafenib or lenvatinib. HCC, hepatocellular carcinoma; CI, confidence interval; HR, hazard ratio; OS, overall survival; PFS, progression-free survival; PS, propensity score.

Multivariate stratified analysis is summarized in Figure 5. In the OS analysis, statin users demonstrated significantly favorable outcomes after adjustment for most covariates, including age, sex, etiology, comorbidities, and comedications. However, statin use was not associated with a lower risk of mortality in patients receiving lenvatinib, patients with underlying PBC, and in SGLT-2 inhibitor and TZD users (Figure 5A). Similar results were observed in PFS analysis (Figure 5B).

### 3.7. Subgroup Analysis according to Sorafenib or Lenvatinib Treatment

For patients receiving sorafenib treatment, the analysis revealed that statin use was an independent factor associated with improved OS and PFS, with an HR of 0.76 (95% CI, 0.71–0.82; *p* < 0.001) for OS. Other factors associated with better OS in this subgroup included living in an urban area, having a history of HTN, cardiovascular disease, cerebrovascular disease, and the use of aspirin, metformin, and sulfonylurea. On the contrary, insulin use was associated with a poorer OS (HR, 1.20; 95%CI, 1.12–1.28; *p* < 0.001; Table S3). PFS analysis yielded similar results, except that older age (>60 years) was associated with poor PFS, and sulfonylurea use did not have a meaningful effect on PFS (Table S4).

In the sorafenib-treated group, both continuous (HR, 0.87; 95% CI, 0.80–0.95; *p* = 0.002) and post-TKI statin use (HR, 0.44; 95% CI, 0.38–0.51; *p* < 0.001) significantly improved OS, along with a history of cerebrovascular and cardiovascular disease and the use of aspirin and antidiabetic medications. In contrast, pre-TKI statin use (HR, 1.28; 95% CI, 1.10–1.49; *p* = 0.002) and insulin use increased the risk of tumor progression (Table S5). For PFS in the sorafenib-treated group, post-TKI statin use was beneficial in reducing the risk of tumor progression (HR, 0.43; 95% CI, 0.38–0.48; *p* < 0.001), along with other factors such as living in urban region and histories of DM, HTN, and vascular disease, as well as the use of aspirin and most antidiabetic medications (Table S6). In the subgroup analysis of patients treated with lenvatinib, the results were less clear, with only insulin use significantly impacting survival outcomes (Tables S7 and S8). When assessing the timing of statin use in the lenvatinib group, post-TKI statin use was associated with favorable outcomes in OS (HR, 0.29; 95% CI, 0.11–0.79; *p* = 0.015), while pre-TKI statin use (HR, 2.27; 95% CI, 1.23–4.22; *p* = 0.009), older age (>60 years), and insulin use were independent risk factors for poor OS (Table S9). For PFS, only post-TKI statin use was beneficial (HR, 0.36; 95% CI, 0.17–0.77; *p* = 0.009) with age >60 years, but insulin use remained a risk factor for tumor progression. Overall, the subgroup analysis showed disparities between the sorafenib and lenvatinib treatment groups, possibly due to the smaller number of participants in the lenvatinib group (Table S10)**.**

## 4. Discussion

This study is noteworthy because it is the first to investigate the potential survival benefits of statins in patients with advanced HCC treated with TKIs using nationwide and multicenter data from the HIRA in Korea. The study begins by acknowledging that statins have previously been associated with a reduced risk of HCC development in patients with chronic liver disease and a decreased risk of tumor recurrence after curative resection in early-stage HCC [10,11,12,13,14,15,16,17,18]. However, statin use is often limited due to safety concerns, particularly in patients with a cirrhotic liver. While a cirrhotic liver makes patients vulnerable to drug-induced liver injury or statin-associated muscle symptoms because of the impaired hepatic metabolism of statins via cytochrome P 268 (CYP)3A4 and reduced multi-drug resistance protein 2 membrane transporter activity, fatal cases are rare [25,26]. The clinical benefits of statins in advanced cirrhosis need to be emphasized beyond potential risks [27,28,29]. Additionally, statins have been shown to induce antitumor effects through various mechanisms, such as apoptosis, the regulation of autophagy, and interaction with the tumor microenvironment [30,31,32,33,34,35,36]. These mechanisms are linked to pathways associated with sorafenib resistance, making statins a potential strategy for overcoming resistance to TKIs.

Simvastatin can re-sensitize sorafenib-resistant HCC cells by inhibiting the hypoxia-inducible 280 factor-1α/PPAR-γ/PKM2 axis and suppressing PKM2-mediated glycolysis, as demonstrated in vitro [37]. Fluvastatin combined with sorafenib induced apoptosis and inhibited hepatic stellate cell activation, showing a synergistic antitumor effect in vivo [38,39,40,41]. Similarly, in in vivo studies, pravastatin combined with sorafenib was shown to further inhibit cancer cell proliferation and exhibit greater efficacy against HCC compared to sorafenib alone [42]. However, the effective role of pravastatin combined with sorafenib in clinical studies is inconsistent [42,43,44,45]. Despite this, statins have been proposed as potential therapeutic agents to overcome sorafenib resistance based on previous experimental and epidemiological evidence. Interestingly, the continuous use of statins, whether initiated before or after TKI treatment, was associated with better survival outcomes, emphasizing the importance of maintaining consistent statin administration even after an HCC diagnosis, consistent with previous findings [46].

The study further explored the impact of the type of statin, revealing that both lipophilic and hydrophilic statins provide survival benefits for patients with advanced HCC undergoing sorafenib treatment, contrary to previous studies favoring lipophilic statins [12,14]. The effects of statins may vary based on their type and underlying liver disease. In cirrhotic livers, intrahepatic angiogenesis, sinusoidal remodeling, and reduced liver perfusion can impair the functionality of CYP enzymes. In particular, the hepatic expression of CYP3A, a key enzyme involved in the metabolism of both sorafenib and lipophilic statins, was found to be reduced in cirrhotic livers. While lipophilic statins passively diffuse through tissues and are metabolized by CYP450, hydrophilic statins are only minimally affected by CYP450, are taken up by hepatic transporters, and can more selectively disrupt lipid metabolism in HCC cells compared to lipophilic statins [47]. In this context, combining hydrophilic statins with sorafenib may offer added benefits for patients with advanced HCC or cirrhosis. In this study, hydrophilic statins exhibited superior survival benefits compared to lipophilic statins, challenging previous preferences for lipophilic statins.

The relationship between various medications and survival outcomes in patients with HCC has been investigated in this study. Aspirin, DPP-4 inhibitors, and metformin are associated with improved outcomes [48,49,50,51], while insulin use and pre-TKI statin use are linked to an increased risk of mortality and tumor recurrence in statin users. In advanced cirrhosis, exogenous insulin is frequently used to prevent hepatoxicity from other diabetes medications. The increased levels of free serum IGF-1 due to insulin resistance in these patients may promote hepatocarcinogenesis via autophagy, leading to a poor prognosis in HCC patients with type II DM [52]. The presence of cardiovascular or cerebrovascular disease in statin users was found to ameliorate all-cause mortality and tumor recurrence, suggesting that statins may have a preventive effect on the survival of HCC patients, particularly those with a history of vascular events.

We emphasize that statins, when coadministered with sorafenib, can offer significant survival benefits in a higher cumulative dose of statins, specifically doses exceeding 730 cDDD for patients with advanced HCC. This study underscores the importance of maintaining statin administration consistently, even after an HCC diagnosis. This approach can be considered as a promising combination therapy, taking into account factors such as cost, effectiveness, and tolerability.

We acknowledge several limitations in this study. First, the study is retrospective in nature and was conducted using a health insurance claims database lacking information on certain risk factors for HCC, such as anthropometric information (including body mass index and waist circumference), laboratory findings assessing hepatic reserve function (e.g., Child-Pugh or modified albumin-bilirubin grade), clinical staging such as the BCLC staging system, and clinical details related to cirrhosis complications (such as ascites, hepatic encephalopathy, and variceal bleeding). Although we acknowledge these missing data could be critical for assessing patient prognosis and outcomes, we were unable to control these potential confounding factors. Second, the study considered all-cause death as the primary outcome, which may not provide insights into liver-related mortality, a more specific and relevant endpoint for HCC patients. Third, although the liver function of patients with advanced HCC may be favorable at the time of statin administration, the progressive decline in the functional reserve volume of the liver and portal HTN due to tumor thrombosis, can lead to a rapid deterioration in liver function, necessitating the discontinuation of statin use. Insufficient details regarding tumor staging, specifically the absence of information on factors such as tumor volume or the extent of portal vein invasion and distant metastasis, could potentially limit conclusive insights of statin dosage and duration in this study. Further exploration on the relationships between the effectiveness and dosages of statins is warranted to offer valuable contributions in real-world practices. Finally, given the distinct underlying factors for HCC in Korea compared to Western countries, the generalizability of our findings to other healthcare systems may be limited. Regional variations in HCC etiology should be considered when interpreting the study results. In our study encompassing patients with HCC from January 1 to December 31, 2020, we could not exclude HCC patients with concurrent COVID-19 infection. The pandemic hindered early HCC detection and treatment due to healthcare access limitations and the risk of complications related to liver cirrhosis and the treatment for HCC, potentially affecting survival outcomes. We acknowledge this as a limitation of our study.

## 5. Conclusions

Our study highlights that statins, when coadministered with sorafenib, can substantially improve OS and PFS in patients with advanced HCC. It emphasizes the importance of continuous statin administration, even after an HCC diagnosis. However, more research, including in vitro studies to understand the molecular mechanisms, and well-designed prospective clinical trials, are needed to establish a solid basis for combining sorafenib and statins as a treatment strategy for advanced HCC.

## Figures and Tables

**Figure 2:**
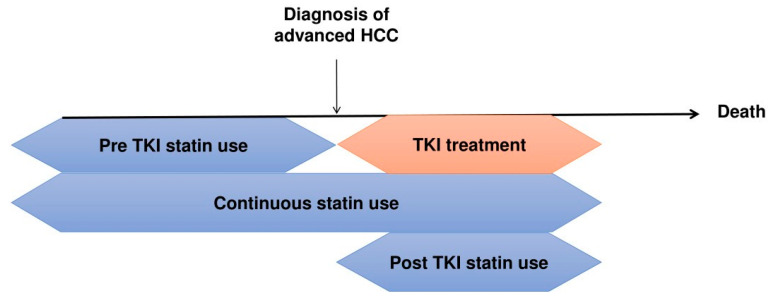
Definitions of statin use regarding the timing of statin administration. HCC, hepatocellular carcinoma; TKI, tyrosine kinase inhibitor.

**Table 1:** Baseline characteristics of statin users and non-users.

	Before PS-Matching			After PS-Matching		
	Non-Users (*n* = 11,062)	Statin Users (*n* = 1534)	*p*-Value	Non-Users (*n* = 6136)	Statin Users (*n* = 1534)	*p*-Value
Age	56.48 (8.28)	59.87 (7.29)	<0.001	59.363 (7.013)	59.870 (7.290)	0.012
Sex, male, No. (%)	9652 (87.3%)	1359 (88.6%)	0.139	5439 (88.6%)	1359 (88.6%)	0.957
Region, No. (%)			0.033			0.004
Rural	1542 (13.9%)	202 (13.2%)		912 (14.9%)	202 (13.2%)	
Gyeong-gi	1721 (15.6%)	242 (15.8%)		906 (14.8%)	242 (15.8%)	
Seoul	5590 (50.5%)	826 (53.8%)		3066 (50.0%)	826 (53.8%)	
Special city	2209 (20.0%)	264 (17.2%)		1252 (20.4%)	264 (17.2%)	
HCC treatment, No. (%)			0.010			0.021
Sorafenib	10,308 (93.2%)	1402 (91.4%)		5713 (93.1%)	1402 (91.4%)	
Lenvatinib	754 (6.8%)	132 (8.6%)		423 (6.9%)	132 (8.6%)	
Statin use pattern, No. (%)			-			-
Pre-TKI use	-	218 (14.2%)		-	218 (14.2%)	
Continuous use from TKI treatment	-	950 (61.9%)		-	950 (61.9%)	
Post-TKI use	-	366 (23.9%)		-	366 (23.9%)	
Etiology, No. (%)						
HBV	9363 (84.6%)	1109 (72.3%)	<0.001	4818 (78.5%)	1109 (72.3%)	<0.001
HCV	1368 (12.4%)	185 (12.1%)	0.732	793 (12.9%)	185 (12.1%)	0.364
Alcoholic	1472 (13.3%)	263 (17.1%)	<0.001	972 (15.8%)	263 (17.1%)	0.214
History of comorbidities						
History of DM, No. (%)	6413 (58.0%)	1303 (84.9%)	<0.001	5163 (84.1%)	1303 (84.9%)	0.442
History of fatty liver, No. (%)	1735 (15.7%)	415 (27.1%)	<0.001	1352 (22.0%)	415 (27.1%)	<0.001
History of cirrhosis, No. (%)	4832 (43.7%)	713 (46.5%)	0.0385	2892(47.1%)	713(46.5%)	0.647
History of HTN, No. (%)	7067 (63.9%)	1397 (91.1%)	<0.001	4433 (72.2%)	1397 (91.1%)	<0.001
History of PBC, No. (%)	52 (0.5%)	17 (1.1%)	0.002	45 (0.7%)	17 (1.1%)	0.143
History of cardiovascular disease, No. (%)	2448 (22.1%)	717 (46.7%)	<0.001	1614 (26.3%)	717 (46.7%)	<0.001
History of cerebrovascular disease, No. (%)	970 (8.8%)	437 (28.5%)	<0.001	718 (11.7%)	437 (28.5%)	<0.001
Antiviral treatment, No. (%)						
HBV treatment	7752 (70.1%)	842 (54.9%)	<0.001	3905 (63.6%)	842 (54.9%)	<0.001
HCV treatment	248 (2.2%)	47 (3.1%)	0.046	793 (12.9%)	185 (12.1%)	0.364
Other medication, No. (%)						
Aspirin use	264 (2.4%)	106 (6.9%)	<0.001	158 (2.6%)	106 (6.9%)	<0.001
DPP-4 inhibitor use	279 (2.5%)	109 (7.1%)	<0.001	241 (3.9%)	109 (7.1%)	<0.001
Insulin use	1717 (15.5%)	285 (18.6%)	0.002	1010 (16.5%)	285 (18.6%)	0.048
Metformin use	229 (2.1%)	54 (3.5%)	<0.001	173 (2.8%)	54 (3.5%)	0.147
SGLT-2 inhibitor use	27 (0.2%)	18 (1.2%)	<0.001	25 (0.4%)	18 (1.2%)	<0.001
Sulfonylurea use	131 (1.2%)	38 (2.5%)	<0.001	109 (1.8%)	38 (2.5%)	0.073
Thiazolidinedione use	43 (0.4%)	21 (1.4%)	<0.001	36 (0.6%)	21 (1.4%)	0.001
Median treatment duration (days)	260.00 (149.00, 498.00)	337.00 (180.00, 708.00)	<0.001	269.00 (152.00, 512.00)	337.00 (180.00, 708.00)	<0.001

Abbreviations: DM, diabetes mellitus; DPP, dipeptidyl peptidase; HBV, hepatitis B virus; HCC, hepatocellular carcinoma; HCV, hepatitis C virus; HTN, hypertension; PBC, primary biliary cholangitis; PS, propensity score; SGLT, sodium-glucose cotransporter; TKI, tyrosine kinase inhibitor.

**Table 2:** Univariate and multivariate Cox regression analysis for OS according to statin use in the entire PS-matched cohort.

Variables	Univariate Analysis			Multivariate Analysis		
	HR	95% CI	*p*-Value	HR	95% CI	*p*-Value
Statin use, yes	0.71	0.66–0.76	<0.001	0.77	0.72–0.82	<0.001
Age, ≥60 years	0.99	0.94–1.04	0.624	1.00	1.00–1.01	0.469
Sex, female	0.93	0.86–1.01	0.080	0.95	0.88–1.03	0.205
Region, urban	0.96	0.89–1.03	0.249			
DM, yes	0.92	0.86–0.99	0.022	0.94	0.87–1.01	0.086
HTN, yes	0.80	0.75–0.85	<0.001	0.87	0.82–0.92	<0.001
Cardiovascular disease, yes	0.86	0.81–0.91	<0.001	0.93	0.88–0.98	0.010
Cerebrovascular disease, yes	0.86	0.80–0.92	<0.001			
Fatty liver, yes	0.90	0.84–0.96	0.001	0.95	0.89–1.01	0.081
Cirrhosis, yes	0.80	0.76-0.84	<0.001			
Aspirin, yes	0.61	0.53–0.70	<0.001	0.64	0.55–0.74	<0.001
DPP-4 inhibitor use, yes	0.85	0.75–0.95	0.006			
Insulin use, yes	1.14	1.07–1.22	<0.001	1.24	1.16–1.33	<0.001
Metformin use, yes	0.75	0.65–0.87	<0.001	0.78	0.67–0.91	0.002
SGLT-2 inhibitor use, yes	0.60	0.41–0.88	0.01	0.67	0.45–0.99	0.044
Sulfonylurea use, yes	0.76	0.63–0.91	0.003	0.81	0.67–0.99	0.037
Thiazolidinedione use, yes	0.65	0.47–0.89	0.007	0.79	0.57–1.09	0.150

Abbreviations: CI, confidence interval; HR, hazard ratio; DM, diabetes mellitus; HTN, hypertension; DPP, dipeptidyl peptidase; OS, overall survival; PS, propensity score; SGLT, sodium-glucose cotransporter.

**Table 3:** Univariate and multivariate Cox regression analysis for PFS according to statin use in PS-matched cohort.

Variables	Univariate Analysis			Multivariate Analysis		
	HR	95% CI	*p*-Value	HR	95% CI	*p*-Value
Statin use, yes	0.72	0.67–0.76	<0.001	0.78	0.74–0.84	<0.001
Age, ≥60 years	1.04	0.99–1.09	0.156	1.01	1.00–1.01	0.006
Sex, female	0.98	0.91–1.06	0.603	1.01	0.94–1.09	0.825
Region, urban	0.88	0.82–0.94	<0.001	0.87	0.81–0.93	<0.001
DM, yes	0.91	0.85–0.98	0.007	0.95	0.88–1.01	0.104
Fatty liver, yes	0.94	0.89–1.00	0.054			
Cirrhosis, yes	0.82	0.79-0.86	<0.001			
HTN, yes	0.80	0.75–0.84	<0.001	0.86	0.81–0.91	<0.001
Cardiovascular disease, yes	0.84	0.79–0.88	<0.001	0.90	0.86–0.96	<0.001
Cerebrovascular disease, yes	0.81	0.76–0.87	<0.001	0.90	0.84–0.97	0.004
Aspirin use, yes	0.57	0.50–0.65	<0.001	0.63	0.55–0.73	<0.001
DPP-4 inhibitor use, yes	0.78	0.70–0.88	<0.001	0.90	0.80–1.01	0.085
Insulin use, yes	1.13	1.06–1.20	<0.001	1.26	1.18–1.35	<0.001
Metformin use, yes	0.68	0.59–0.78	<0.001	0.72	0.62–0.84	<0.001
SGLT-2 inhibitor use, yes	0.70	0.50–0.98	0.038	0.77	0.54–1.07	0.123
Sulfonylurea use, yes	0.73	0.61–0.86	<0.001	0.83	0.69–1.00	0.049
Thiazolidinedione use, yes	0.64	0.49–0.85	0.002	0.80	0.60–1.06	0.114

Abbreviations: CI, confidence interval; HR, hazard ratio; DM, diabetes mellitus; HTN, hypertension; DPP, dipeptidyl peptidase; PFS, progression free survival; PS, propensity score; SGLT, sodium-glucose cotransporter.

**Table 4:** Univariate and multivariate Cox regression analysis for OS according to statin use pattern.

Variables	Univariate Analysis			Multivariate Analysis		
	HR	95% CI	*p*-Value	HR	95% CI	*p*-Value
Statin use pattern						
Non-user	reference			reference		
Pre-TKI use	1.31	1.13–1.52	<0.001	1.33	1.14–1.54	<0.001
Continuous use from TKI treatment	0.80	0.74–0.87	<0.001	0.87	0.80–0.95	0.002
Post-TKI use	0.40	0.35–0.46	<0.001	0.43	0.38–0.50	<0.001
Age, ≥60 years	0.99	0.94–1.04	0.624	1.00	1.00–1.00	0.767
Sex, female	0.93	0.86–1.01	0.080	0.95	0.88–1.03	0.246
Region, urban	0.96	0.89–1.03	0.249			
DM, yes	0.92	0.86–0.99	0.022	0.93	0.87–1.00	0.048
Fatty liver, yes	0.90	0.84–0.96	<0.001	0.94	0.88–1.00	0.050
Cirrhosis, yes	0.80	0.76-0.84	<0.001			
HTN, yes	0.80	0.75–0.85	<0.001	0.87	0.82–0.92	<0.001
Cardiovascular disease, yes	0.86	0.81–0.91	<0.001	0.93	0.88–0.99	0.016
Cerebrovascular disease, yes	0.86	0.80–0.92	<0.001			
Aspirin use, yes	0.61	0.53–0.70	<0.001	0.69	0.59–0.79	<0.001
DPP-4 inhibitor use, yes	0.85	0.75–0.95	0.006	0.90	0.80–1.03	0.119
Insulin use, yes	1.14	1.07–1.22	<0.001	1.23	1.15–1.32	<0.001
Metformin use, yes	0.68	0.59–0.78	<0.001	0.83	0.71–0.97	0.022
SGLT-2 inhibitor use, yes	0.60	0.41–0.88	0.010	0.66	0.45–0.98	0.038
Sulfonylurea use, yes	0.76	0.63–0.91	0.003	0.79	0.65–0.96	0.020
Thiazolidinedione use, yes	0.65	0.47–0.89	0.007			

Abbreviations: CI, confidence interval; HR, hazard ratio; DM, diabetes mellitus; HTN, hypertension; DPP, dipeptidyl peptidase; OS, overall survival; SGLT, sodium-glucose cotransporter; TKI, tyrosine kinase inhibitor.

**Table 5:** Univariate and multivariate Cox regression analysis for PFS according to statin use pattern.

Variables	Univariate Analysis			Multivariate Analysis		
	HR	95% CI	*p*-Value	HR	95% CI	*p*-Value
Statin use pattern						
Non-user	reference			reference		
Pre-TKI use	1.53	1.33–1.77	<0.001	1.56	1.35–1.80	<0.001
Continuous use from TKI treatment	0.86	0.80–0.93	<0.001	0.94	0.87–1.02	0.122
Post-TKI use	0.38	0.34–0.43	<0.001	0.42	0.38–0.48	<0.001
Age ≥60 years	1.04	0.99–1.09	0.156	1.00	1.00–1.01	0.032
Sex, female	0.98	0.91–1.06	0.603	1.02	0.95–1.10	0.571
Region, urban	0.88	0.82–0.94	<0.001	0.86	0.80–0.92	<0.001
DM, yes	0.91	0.85–0.98	0.007	0.92	0.86–0.99	0.023
Fatty liver, yes	0.94	0.89–1.00	0.054			
Cirrhosis, yes	0.82	0.79-0.86	<0.001			
HTN, yes	0.80	0.75–0.84	<0.001	0.86	0.81–0.91	<0.001
Cardiovascular disease, yes	0.84	0.79–0.88	<0.001	0.91	0.86–0.96	0.001
Cerebrovascular disease, yes	0.81	0.76–0.87	<0.001	0.91	0.85–0.98	0.012
Aspirin use, yes	0.57	0.50–0.65	<0.001	0.68	0.59–0.77	<0.001
DPP-4 inhibitor use, yes	0.78	0.70–0.88	<0.001	0.88	0.78–0.99	0.035
Insulin use, yes	1.13	1.06–1.20	<0.001	1.25	1.17–1.33	<0.001
Metformin use, yes	0.68	0.59–0.78	<0.001	0.75	0.64–0.87	<0.001
SGLT-2 inhibitor use, yes	0.70	0.50–0.98	0.038	0.74	0.53–1.03	0.077
Sulfonylurea use, yes	0.73	0.61–0.86	<0.001	0.81	0.67–0.97	0.022
Thiazolidinedione use, yes	0.64	0.49–0.85	0.002			

Abbreviations: CI, confidence interval; HR, hazard ratio; DM, diabetes mellitus; HTN, hypertension; DPP, dipeptidyl peptidase; PFS, progression free survival; SGLT, sodium-glucose cotransporter; TKI, tyrosine kinase inhibitor.

## Data Availability

The data presented in this study are available on request from the corresponding author.

## Supplementary Materials

The following supporting information can be downloaded at: https://www.mdpi.com/article/10.3390/cancers16020249/s1. Table S1. Survival outcomes according to statin type; Table S2. Survival outcomes according to statin dose; Table S3. Univariate and multivariate Cox regression analysis for OS treated with sorafenib according to statin use in a PS-matched cohort; Table S4. Univariate and multivariate Cox regression analysis for PFS treated with sorafenib according to statin use in a PS-matched cohort; Table S5. Univariate and multivariate Cox regression analysis for OS treated with sorafenib according to statin use pattern in a PS-matched cohort; Table S6. Univariate and multivariate Cox regression analysis for PFS treated with sorafenib according to statin use pattern in a PS-matched cohort; Table S7. Univariate and multivariate Cox regression analysis for OS treated with lenvatinib according to statin use in a PS-matched cohort; Table S8. Univariate and multivariate Cox regression analysis for PFS treated with lenvatinib according to statin use in a PS-matched cohort; Table S9. Univariate and multivariate Cox regression analysis for OS treated with lenvatinib according to statin use pattern in a PS-matched cohort; Table S10. Univariate and multivariate Cox regression analysis for PFS treated with lenvatinib according to statin use pattern in a PS-matched cohort; Figure S1. Kaplan–Meier curves of OS and PFS according to statin use and statin type in the sorafenib subgroup; Figure S2. Kaplan–Meier curves of OS and PFS according to statin use and statin type in the lenvatinib subgroup.
