# Supplementary material for: The Impact of Statins on the Survival of Patients with Advanced Hepatocellular Carcinoma Treated with Sorafenib or Lenvatinib
Source: Cancers (Basel). 2024 Jan 5;16(2):249. doi: 10.3390/cancers16020249 (PMC10813381; doi:10.3390/cancers16020249)
Supplement: Supplementary file 1 [file cancers-16-00249-s001.zip › cancers-2782954-supplementary.pdf]

Supplementary Materials: The Impact of Statins on the Survival of Patients with Advanced Hepatocellular Carcinoma Treated with Sorafenib or Lenvatinib

Ji Eun Han, Jisu Kim, Jae Youn Cheong, Soon Sun Kim, Sun Gyo Lim, Min Jae Yang, Choong-Kyun Noh, Gil Ho Lee, Jung Woo Eun, Bumhee Park and Hyo Jung Cho

Table S1. Survival outcomes according to statin type.

Outcomes	Lipophilic statin					Hydrophilic statin				
	No	Yes				No	Yes			
		HR	95% CI		P-value		HR	95% CI		P-value
PFS (unadjusted HR, 95% CI)	ref	0.74	0.69	0.80	<0.001	ref	0.63	0.57	0.69	<0.001
OS (unadjusted HR, 95% CI)	ref	0.75	0.69	0.81	<0.001	ref	0.59	0.53	0.66	<0.001

Abbreviations: CI, confidence interval; HR, hazard ratio; OS, overall survival; PFS, progression-free survival.

Table S2. Survival outcomes according to statin dose.

Outcomes	<181 cDDD	181–365 cDDD				366–730 cDDD				731–1,095 cDDD				≥1,096 cDDD			
	ref	HR	95% CI		P-value	HR	95% CI		P-value	HR	95% CI		P-value	HR	95% CI		P-value
PFS (unadjusted HR, 95% CI)		1.14	0.96	1.37	0.1385	0.97	0.81	1.15	0.697	0.56	0.46	0.69	<0.0001	0.34	0.28	0.41	<0.001
OS (unadjusted HR, 95% CI)		1.19	0.99	1.44	0.0646	0.92	0.77	1.11	0.397	0.60	0.48	0.75	<0.0001	0.34	0.28	0.42	<0.001

Abbreviations: cDDD, cumulative defined daily doses; CI, confidence interval; HR, hazard ratio; OS, overall survival; PFS, progression-free survival.

Table S3. Univariate and multivariate cox regression analysis for OS treated with sorafenib according to statin use in a PS-matched cohort.

Variables	Univariate analysis			Multivariate analysis		
	HR	95% CI	P-value	HR	95% CI	P-value
Statin use, yes	0.71	0.66–0.76	<0.001	0.76	0.71–0.82	<0.001
Age, ≥60 yr	0.99	0.94–1.04	0.624	1.00	1.00–1.01	0.245
Sex, female	0.93	0.86–1.01	0.080	0.95	0.87–1.02	0.174
Region, urban	0.96	0.89–1.03	0.249	0.86	0.81–0.92	<0.001
DM, yes	0.92	0.86–0.99	0.022	0.94	0.87–1.01	0.085
Fatty liver, yes	0.90	0.84–0.96	0.001	0.95	0.89–1.01	0.100
HTN, yes	0.80	0.75–0.85	<0.001	0.87	0.82–0.92	<0.001
Cardiovascular disease, yes	0.86	0.81–0.91	<0.001	0.93	0.88–0.98	0.010
Cerebrovascular disease, yes	0.86	0.80–0.92	<0.001			
Aspirin use, yes	0.61	0.53–0.70	<0.001	0.63	0.55–0.73	<0.001
DPP-4 inhibitor use, yes	0.85	0.75–0.95	0.006	0.89	0.79–1.00	0.060
Insulin use, yes	1.14	1.07–1.22	<0.001	1.20	1.12–1.28	<0.001
Metformin use, yes	0.75	0.65–0.87	<0.001	0.77	0.66–0.91	0.002
SGLT-2 inhibitor use, yes	0.60	0.41–0.88	0.010	0.69	0.47–1.02	0.062
Sulfonylurea use, yes	0.76	0.63–0.91	0.003	0.82	0.67–0.99	0.044
Thiazolidinedione use, yes	0.65	0.47–0.89	0.007	0.76	0.55–1.05	0.099

Abbreviations: CI, confidence interval; HR, hazard ratio; DM, diabetes mellitus; HTN, hypertension; DPP, dipeptidyl peptidase; OS, overall survival; PS, propensity score; SGLT, sodium-glucose co-transporter.

Table S4. Univariate and multivariate cox regression analysis for PFS treated with sorafenib according to statin use in a PS-matched cohort.

Variables	Univariate analysis			Multivariate analysis		
	HR	95% CI	P-value	HR	95% CI	P-value
Statin use, yes	0.72	0.67–0.76	<0.001	0.78	0.73–0.84	<0.001
Age, ≥60 yr	1.04	0.99–1.09	0.156	1.01	1.00–1.01	0.002
Sex, female	0.98	0.91–1.06	0.603	1.01	0.93–1.09	0.878
Region, urban	0.88	0.82–0.94	<0.001	0.86	0.81–0.92	<0.001
DM, yes	0.91	0.85–0.98	0.007	0.94	0.88–1.01	0.073
Fatty liver, yes	0.94	0.89–1.00	0.054			
HTN, yes	0.80	0.75–0.84	<0.001	0.86	0.81–0.91	<0.001
Cardiovascular disease, yes	0.84	0.79–0.88	<0.001	0.90	0.85–0.95	<0.001
Cerebrovascular disease, yes	0.81	0.76–0.87	<0.001	0.91	0.85–0.98	0.008
Aspirin use, yes	0.57	0.50–0.65	<0.001	0.63	0.55–0.72	<0.001
DPP-4 inhibitor use, yes	0.78	0.70–0.88	<0.001	0.89	0.79–1.00	0.060
Insulin use, yes	1.13	1.06–1.20	<0.001	1.25	1.17–1.33	<0.001
Metformin use, yes	0.68	0.59–0.78	<0.001	0.72	0.61–0.84	<0.001
SGLT-2 inhibitor use, yes	0.70	0.50–0.98	0.038			
Sulfonylurea use, yes	0.73	0.61–0.86	<0.001	0.84	0.70–1.01	0.064
Thiazolidinedione use, yes	0.64	0.49–0.85	0.002	0.76	0.57–1.01	0.063

Abbreviations: CI, confidence interval; HR, hazard ratio; DM, diabetes mellitus; HTN, hypertension; DPP, dipeptidyl peptidase; PFS, progression free survival; PS, propensity score; SGLT, sodium-glucose cotransporter; TKI, tyrosine kinase inhibitor.

Table S5. Univariate and multivariate cox regression analysis for OS treated with sorafenib according to statin use pattern in a PS-matched cohort.

Variables	Univariate analysis				Multivariate analysis			
	HR	95% CI		P-value	HR	95% CI		P-value
Statin use pattern								
Non-user		Reference				Reference		
Pre-TKI use	1.31	1.13–		<0.001	1.28	1.10–	1.49	0.002
Continuous use from TKI treatment	0.80	0.74–	0.87	<0.001	0.87	0.80–	0.95	0.002
post-TKI use	0.40	0.35–	0.46	<0.001	0.44	0.38–	0.51	<0.001
Age, ≥60 yr	0.99	0.94–	1.04	0.624	1.00	1.00–	1.01	0.444
Sex, female	0.99	0.94–	1.04	0.624	0.95	0.88–	1.03	0.218
Region, urban	0.96	0.89–	1.03	0.249				
DM, yes	0.92	0.86–	0.99	0.022	0.93	0.87–	1.00	0.053
Fatty liver, yes	0.90	0.84–	0.96	<0.001	0.94	0.88–	1.00	0.058
HTN, yes	0.80	0.75–	0.85	<0.001	0.94	0.88–	1.00	0.058
Cardiovascular disease, yes	0.86	0.81–	0.91	<0.001	0.93	0.88–	0.99	0.015
Cerebrovascular disease, yes	0.86	0.80–	0.92	<0.001				
Aspirin use, yes	0.61	0.53–	0.70	<0.001	0.67	0.58–	0.78	<0.001
DPP-4 inhibitor use, yes	0.85	0.75–	0.95	0.006	0.90	0.79–	1.02	0.100
Insulin use, yes	1.14	1.07–	1.22	<0.001	1.19	1.11–	1.27	<0.001
Metformin use, yes	0.75	0.65	0.87	<0.001	0.82	0.70–	0.96	0.016
SGLT-2 inhibitor use, yes	0.60	0.41	0.88	0.001	0.68	0.46–	1.00	0.048
Sulfonylurea use, yes	0.76	0.63	0.91	0.003	0.80	0.66–	0.97	0.025

Thiazolidinedione use, yes	0.65	0.47	0.89	0.007

Abbreviations: CI, confidence interval; HR, hazard ratio; DM, diabetes mellitus; HTN, hypertension; DPP, dipeptidyl peptidase; OS, overall survival; PS, propensity score; SGLT, sodium-glucose cotransporter; TKI, tyrosine kinase inhibitor.

Table S6. Univariate and multivariate cox regression analysis for PFS treated with sorafenib according to statin use pattern in a PS-matched cohort.

Variables	Univariate analysis				Multivariate analysis			
	HR	95% CI		P-value	HR	95% CI		P-value
Statin use pattern								
Non-user		Reference				Reference		
Pre-TKI use	1.53	1.33	1.77	<0.001	1.56	1.35	1.81	<0.001
Continuous use from TKI treatment	0.86	0.80	0.93	<0.001	0.94	0.87	1.02	0.125
post-TKI use	0.38	0.34	0.43	<0.001	0.43	0.38	0.48	<0.001
Age, ≥60 yr	1.04	0.99	1.09	0.156	1.00	1.00	1.01	0.015
Sex, female	0.98	0.91	1.06	0.603	1.02	0.95	1.10	0.5962
Region, urban	0.88	0.82	0.94	<0.001	0.86	0.80	0.92	<0.001
DM, yes	0.91	0.85	0.98	0.007	0.92	0.86	0.98	0.017
Fatty liver, yes	0.94	0.89	1.00	0.054				
HTN, yes	0.80	0.75	0.84	<0.001	0.86	0.81	0.91	<0.001
Cardiovascular disease, yes	0.84	0.79	0.88	<0.001	0.90	0.85	0.95	0.0002
Cerebrovascular disease, yes	0.81	0.76	0.87	<0.001	0.92	0.86	0.99	0.027
Aspirin use, yes	0.57	0.50	0.65	<0.001	0.67	0.58	0.77	<0.001
DPP-4 inhibitor use, yes	0.78	0.70	0.88	<0.001	0.87	0.77	0.99	0.028
Insulin use, yes	1.13	1.06	1.20	<0.001	1.23	1.15	1.32	<0.001
Metformin use, yes	0.68	0.59	0.78	<0.001	0.74	0.64	0.87	<0.001
SGLT-2 inhibitor use, yes	0.70	0.50	0.98	0.038				
Sulfonylurea use, yes	0.73	0.61	0.86	<0.001	0.83	0.69	1.00	0.048
Thiazolidinedione use, yes	0.64	0.49	0.85	0.002	0.79	0.59	1.06	0.114

Abbreviations: CI, confidence interval; HR, hazard ratio; DM, diabetes mellitus; HTN, hypertension; DPP, dipeptidyl peptidase; PFS, progression free survival; PS, propensity score; SGLT, sodium-glucose cotransporter; TKI, tyrosine kinase inhibitor.

Table S7. Univariate and multivariate cox regression analysis for OS treated with lenvatinib according to statin use in a PS-matched cohort.

Variables	Univariate analysis				Multivariate analysis			
	HR	95% CI		P-value	HR	95% CI		P-value
Statin use, yes	0.71	0.66	0.76	<0.001	0.91	0.65	1.28	0.581
Age, ≥60 yr	0.99	0.94	1.04	0.624	0.99	0.96	1.01	0.152
Sex, female	0.93	0.86	1.01	0.080	1.24	0.76	2.03	0.387
Region, urban	0.96	0.89	1.03	0.249				
Etiology								
HBV	1.03	0.97	1.10	0.310				
HCV	0.97	0.90	1.05	0.468				
Alcoholic	0.97	0.91	1.04	0.443	0.71	0.46	1.10	0.123
DM, yes	0.92	0.86	0.99	0.022				
Fatty liver, yes	0.90	0.84	0.96	0.001				
HTN, yes	0.80	0.75	0.85	<0.001				
Cardiovascular disease, yes	0.86	0.81	0.91	<0.001				
Cerebrovascular disease, yes	0.86	0.80	0.92	<0.001				
Aspirin use, yes	0.61	0.53	0.70	<0.001				

DPP-4 inhibitor use, yes	0.85	0.75	0.95	0.006				
Insulin use, yes	1.14	1.07	1.22	<0.001	2.49	1.79	3.44	<0.001
Metformin use, yes	0.75	0.65	0.87	<0.001				
SGLT-2 inhibitor use, yes	0.60	0.41	0.88	0.010				
Sulfonylurea use, yes	0.76	0.63	0.91	0.003				
Thiazolidinedione use, yes	0.65	0.47	0.89	0.007				

Abbreviations: CI, confidence interval; HR, hazard ratio; DM, diabetes mellitus; HBV, hepatitis B virus; HCV, hepatitis C virus; HTN, hypertension; DPP, dipeptidyl peptidase; OS, overall survival; PS, propensity score; SGLT, sodium-glucose cotransporter; TKI, tyrosine kinase inhibitor.

Table S8. Univariate and multivariate cox regression analysis for PFS treated with lenvatinib according to statin use in a PS-matched cohort.

Variables	Univariate analysis				Multivariate analysis			
	HR	95% CI		P-value	HR	95% CI		P-value
Statin use, yes	0.72	0.67	0.76	<0.001	0.81	0.59	1.11	0.184
Age, ≥60 yr	1.04	0.99	1.04	0.156	0.98	0.97	1.00	0.084
Sex, female	0.98	0.91	1.06	0.603	1.20	0.78	1.86	0.403
Region, urban	0.88	0.82	0.94	<0.001				
Etiology								
HBV	0.95	0.90	1.01	0.103				
HCV	0.92	0.86	0.99	0.034				
Alcoholic	1.03	0.96	1.10	0.373				
DM, yes	0.91	0.85	0.98	0.001				
Fatty liver, yes	0.94	0.89	1.00	0.054				
HTN, yes	0.80	0.75	0.84	<0.001				
Cardiovascular disease, yes	0.84	0.79	0.88	<0.001				
Cerebrovascular disease, yes	0.81	0.76	0.87	<0.001				
Aspirin use, yes	0.57	0.50	0.65	<0.001				
DPP-4 inhibitor use, yes	0.78	0.70	0.88	<0.001				
Insulin use, yes	1.13	1.06	1.20	<0.001	1.97	1.44	2.70	<0.001
Metformin use, yes	0.68	0.59	0.78	<0.001				
SGLT-2 inhibitor use, yes	0.70	0.50	0.98	0.038				
Sulfonylurea use, yes	0.73	0.61	0.86	<0.001				
Thiazolidinedione use, yes	0.64	0.49	0.85	0.002				

Abbreviations: CI, confidence interval; HR, hazard ratio; DM, diabetes mellitus; HBV, hepatitis B virus; HCV, hepatitis C virus; HTN, hypertension; DPP, dipeptidyl peptidase; PFS, progression free survival; PS, propensity score; SGLT, sodium-glucose cotransporter.

Table S9. Univariate and multivariate cox regression analysis for OS treated with lenvatinib according to statin use pattern in a PS-matched cohort.

Variables	Univariate analysis				Multivariate analysis			
	HR	95% CI		P-value	HR	95% CI		P-value
Statin use pattern								
Non-user		Reference				Reference		
Pre-TKI use	1.31	1.13	1.52	<0.001	2.27	1.23	4.22	0.009
Continuous use from TKI treatment	0.80	0.74	0.87	<0.001	0.95	0.63	1.42	0.793
Post-TKI use	0.40	0.35	0.46	<0.001	0.29	0.11	0.79	0.015
Age, ≥60 yr	0.99	0.94	1.04	0.624	0.98	0.96	1.00	0.034
Sex, female	0.93	0.86	1.01	0.080	1.23	0.75	2.00	0.409
Region, urban	0.96	0.89	1.03	0.249				
Etiology								

HBV	1.03	0.97	1.10	0.310				
HCV	0.97	0.90	1.05	0.468				
Alcoholic	0.97	0.91	1.04	0.443	0.62	0.41	0.95	0.029
DM, yes	0.92	0.86	0.99	0.022				
Fatty liver, yes	0.90	0.84	0.96	<0.001				
HTN, yes	0.80	0.75	0.85	<0.001				
Cardiovascular disease, yes	0.86	0.81	0.91	<0.001				
Cerebrovascular disease, yes	0.86	0.80	0.92	<0.001				
Aspirin use, yes	0.61	0.53	0.70	<0.001				
DPP-4 inhibitor use, yes	0.85	0.75	0.95	0.006				
Insulin use, yes	1.14	1.07	1.22	<0.001	2.30	1.66	3.18	<0.001
Metformin use, yes	0.75	0.65	0.87	<0.001				
SGLT-2 inhibitor use, yes	0.60	0.41	0.88	0.010				
Sulfonylurea use, yes	0.76	0.63	0.91	0.003				
Thiazolidinedione use, yes	0.65	0.47	0.89	0.007				

Abbreviations: CI, confidence interval; HR, hazard ratio; DM, diabetes mellitus; HBV, hepatitis B virus; HCV, hepatitis C virus; HTN, hypertension; DPP, dipeptidyl peptidase; OS, overall survival; PS, propensity score; SGLT, sodium-glucose cotransporter; TKI, tyrosine kinase inhibitor.

Table S10. Univariate and multivariate cox regression analysis for the PFS treated with lenvatinib according to the statin use pattern in a PS-matched cohort.

**Variables**	**Univariate analysis**				**Multivariate analysis**			
	**HR**	**95% CI**		**P-value**	**HR**	**95% CI**		**P-value**
Statin use pattern								
Non-user	Reference				Reference			
Pre-TKI use	1.53	1.33	1.77	<0.001	1.25	0.65	2.40	0.496
Continuous use from TKI treatment	0.86	0.80	0.93	<0.001	0.95	0.65	1.37	0.768
Post-TKI use	0.38	0.34	0.43	<0.001	0.36	0.17	0.77	0.009
Age, ≥60 yr	1.04	0.99	1.09	0.156	0.98	0.96	1.00	0.048
Sex, female	0.98	0.91	1.06	0.603	1.16	0.75	1.81	0.499
Region, urban	0.88	0.82	0.94	<0.001				
Etiology								
HBV	0.95	0.90	1.01	0.103				
HCV	0.92	0.86	0.99	0.034				
Alcoholic	1.03	0.96	1.10	0.373				
DM, yes	0.91	0.85	0.98	0.007				
Fatty liver, yes	0.94	0.89	1.00	0.054				
HTN, yes	0.80	0.75	0.84	<0.001				
Cardiovascular disease, yes	0.84	0.79	0.88	<0.001				
Cerebrovascular disease, yes	0.81	0.76	0.87	<0.001				
Aspirin use, yes	0.57	0.50	0.65	<0.001				
DPP-4 inhibitor use, yes	0.78	0.70	0.88	<0.001				
Insulin use, yes	1.13	1.06	1.20	<0.001	1.93	1.40	2.65	<0.001
Metformin use, yes	0.68	0.59	0.78	<0.001				
SGLT-2 inhibitor use, yes	0.70	0.50	0.98	0.038				
Sulfonylurea use, yes	0.73	0.61	0.86	<0.001				
Thiazolidinedione use, yes	0.64	0.49	0.85	0.002				

Abbreviations: Abbreviations: CI, confidence interval; HR, hazard ratio; DM, diabetes mellitus; HBV, hepatitis B virus; HCV, hepatitis C virus; HTN, hypertension; DPP, dipeptidyl peptidase; PFS, progression free survival; PS, propensity score; SGLT, sodium-glucose cotransporter; TKI, tyrosine kinase inhibitor.

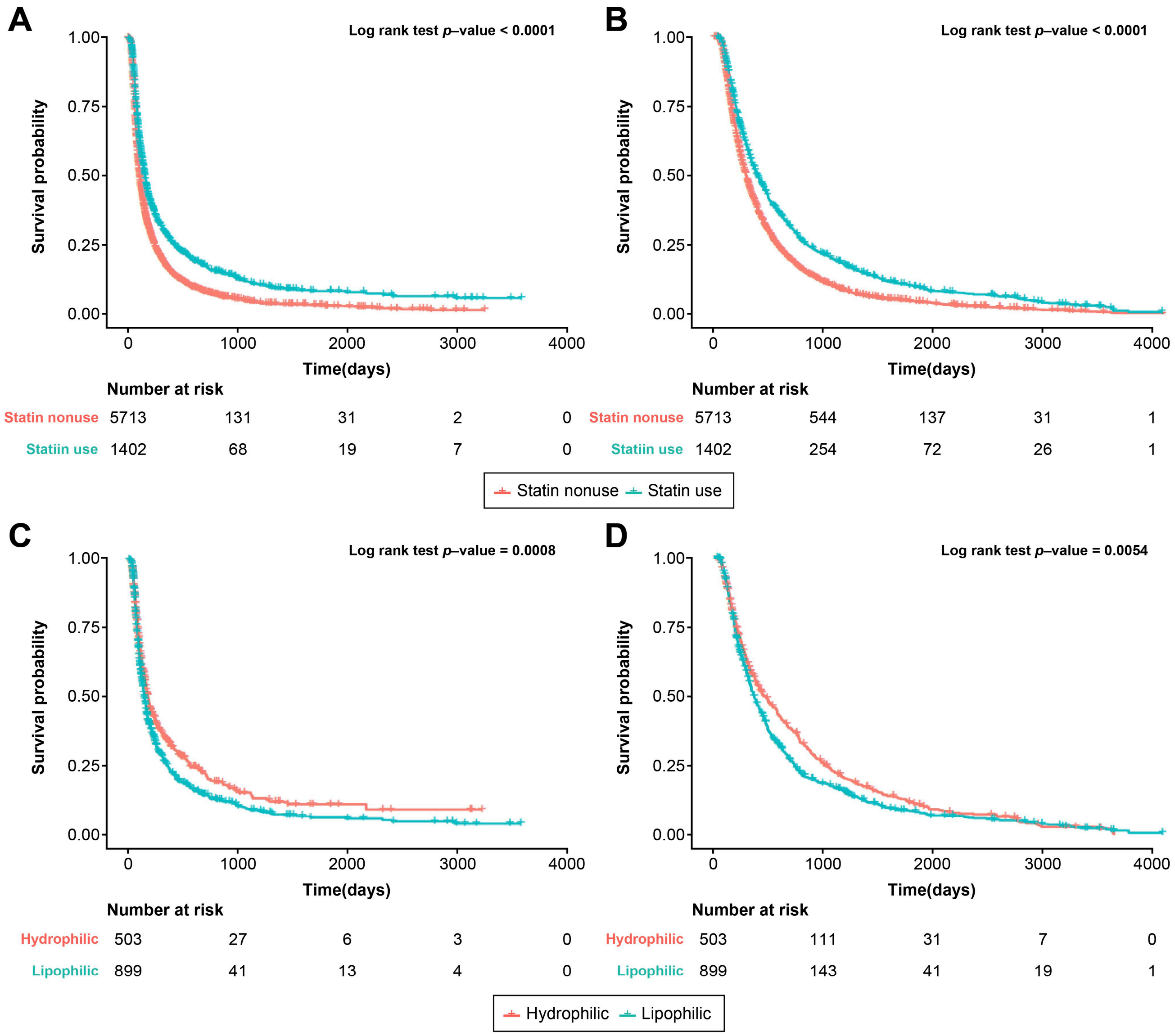

Figure S1. Kaplan–Meier curves of OS and PFS according to statin use and statin type in the sorafenib subgroup. (a) Comparison of OS between statin users and non-users. (b) Comparison of PFS between statin users and non-users. (c) Comparison of OS according to statin type (hydrophilic vs lipophilic). (d) Comparison of PFS according to statin type (hydrophilic vs lipophilic). OS; overall survival, PFS; progression-free survival.

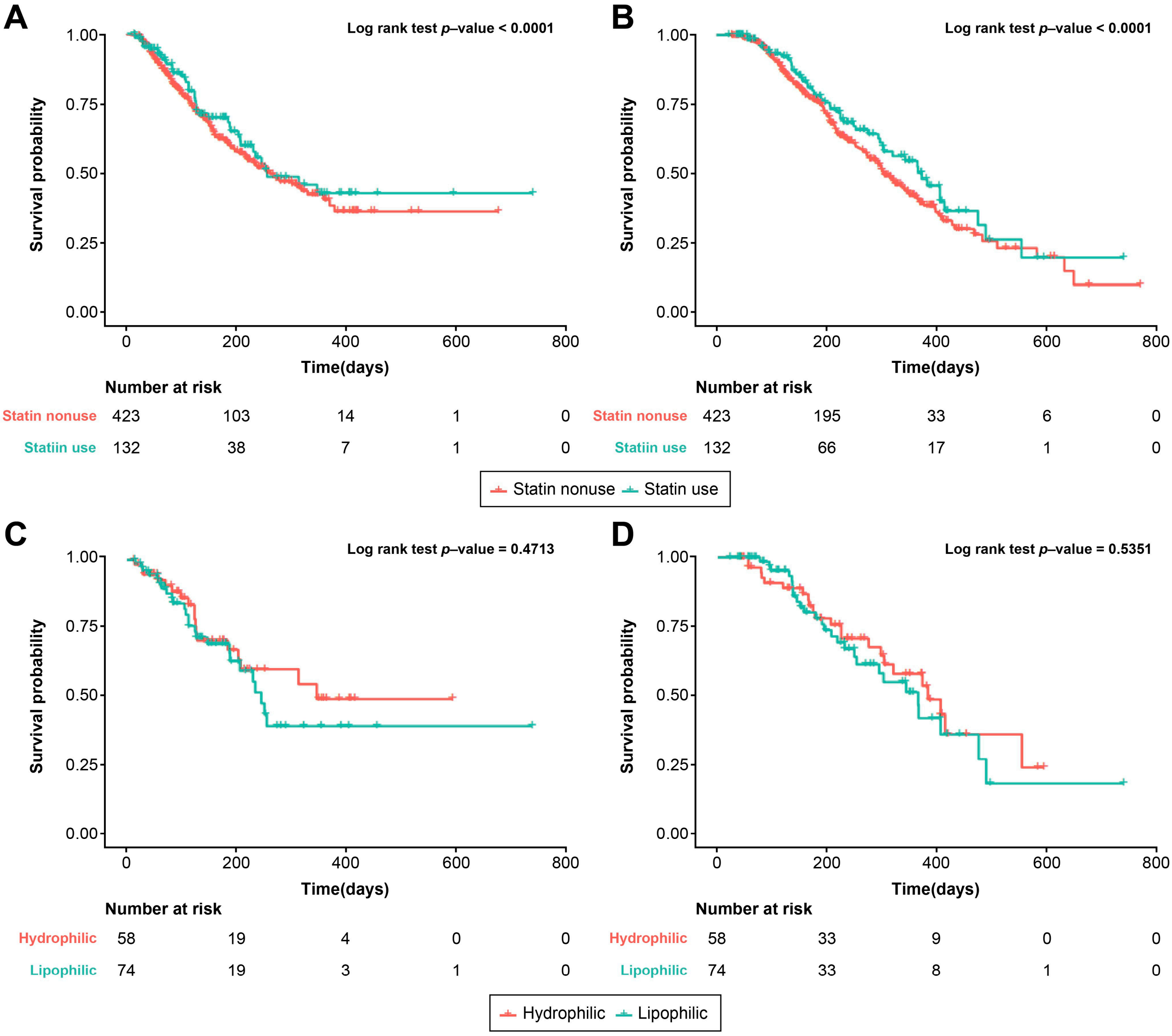

Figure S2. Kaplan–Meier curves of OS and PFS according to statin use and statin type in the lenvatinib subgroup. (a) Comparison of OS between statin users and non-users. (b) Comparison of PFS between statin users and non-users. (c) Comparison of OS according to statin type (hydrophilic vs lipophilic). (d) Comparison of PFS according to statin type (hydrophilic vs lipophilic). OS; overall survival, PFS; progression-free survival.
